# Double-bundle anterior cruciate ligament reconstruction is superior to single-bundle reconstruction in terms of revision frequency: a study of 22,460 patients from the Swedish National Knee Ligament Register

**DOI:** 10.1007/s00167-016-4387-4

**Published:** 2016-11-23

**Authors:** Eleonor Svantesson, David Sundemo, Eric Hamrin Senorski, Eduard Alentorn-Geli, Volker Musahl, Freddie H. Fu, Neel Desai, Anders Stålman, Kristian Samuelsson

**Affiliations:** 1 Department of Orthopaedics, Sahlgrenska University Hospital, 431 80 Mölndal, Sweden; 2 Department of Orthopaedics, Institute of Clinical Sciences, The Sahlgrenska Academy, University of Gothenburg, Gothenburg, Sweden; 3 Department of Orthopaedic Surgery, Mayo Clinic, Rochester, MN USA; 4 Department of Orthopaedic Surgery, University of Pittsburgh, Pittsburgh, PA USA; 5 Department of Molecular Medicine and Surgery, Stockholm Sports Trauma Research Center, Karolinska Institutet, Stockholm, Sweden

**Keywords:** Anterior cruciate ligament, ACL, Reconstruction, Revision, Single-bundle, Double-bundle, Revision, Surgery, Anatomic, Checklist

## Abstract

**Purpose:**

Studies comparing single- and double-bundle anterior cruciate ligament (ACL) reconstructions often include a combined analysis of anatomic and non-anatomic techniques. The purpose of this study was to compare the revision rates between single- and double-bundle ACL reconstructions in the Swedish National Knee Ligament Register with regard to surgical variables as determined by the anatomic ACL reconstruction scoring checklist (AARSC).

**Methods:**

Patients from the Swedish National Knee Ligament Register who underwent either single- or double-bundle ACL reconstruction with hamstring tendon autograft during the period 2007–2014 were included. The follow-up period started with primary ACL reconstruction, and the outcome measure was set as revision surgery. An online questionnaire based on the items of the AARSC was used to determine the surgical technique implemented in the single-bundle procedures. These were organized into subgroups based on surgical variables, and the revision rates were compared with the double-bundle ACL reconstruction. Hazard ratios (HR) with 95% confidence interval (CI) was calculated and adjusted for confounders by Cox regression.

**Results:**

A total of 22,460 patients were included in the study, of which 21,846 were single-bundle and 614 were double-bundle ACL reconstruction. Double-bundle ACL reconstruction had a revision frequency of 2.0% (*n* = 12) and single-bundle 3.2% (*n* = 689). Single-bundle reconstruction had an increased risk of revision surgery compared with double-bundle [adjusted HR 1.98 (95% CI 1.12–3.51), *p* = 0.019]. The subgroup analysis showed a significantly increased risk of revision surgery in patients undergoing single-bundle with anatomic technique using transportal drilling [adjusted HR 2.51 (95% CI 1.39–4.54), *p* = 0.002] compared with double-bundle ACL reconstruction. Utilizing a more complete anatomic technique according to the AARSC lowered the hazard rate considerably when transportal drilling was performed but still resulted in significantly increased risk of revision surgery compared with double-bundle ACL reconstruction [adjusted HR 1.87 (95% CI 1.04–3.38), *p* = 0.037].

**Conclusions:**

Double-bundle ACL reconstruction is associated with a lower risk of revision surgery than single-bundle ACL reconstruction. Single-bundle procedures performed using transportal femoral drilling technique had significantly higher risk of revision surgery compared with double-bundle. However, a reference reconstruction with transportal drilling defined as a more complete anatomic reconstruction reduces the risk of revision surgery considerably.

**Level of evidence:**

III.

## Introduction

Recent years’ knowledge about knee anatomy and kinematics has increased the interest in performing anatomic anterior cruciate ligament (ACL) reconstruction. The ACL consists of at least two distinct functional bundles: the anteromedial bundle and the posterolateral bundle [7]. An anatomic double-bundle ACL reconstruction is therefore close to the native anatomy. Currently though, the single-bundle ACL reconstruction is the most utilized method [22], even though double-bundle reconstruction increases the rotational stability [3, 22, 24, 28, 29]. Several meta-analyses, systematic reviews and a Cochrane review have confirmed superior knee-stability provided by the double-bundle reconstruction, and a Cochrane review showed a trend towards lower re-rupture frequency in favour of the double-bundle reconstruction [3, 13, 17, 22]. However, only two randomized controlled trials report that a double-bundle reconstruction reduces the risk of graft failure [20, 21].

Results from two recent register studies showed that the revision rates among single-bundle reconstructions differ depending on the surgical techniques implemented [4, 18]. The studies concluded that single-bundle reconstructions performed using transportal (TP) drilling had an increased risk of revision surgery; however, the results also indicated that there was a learning curve and that a reference reconstruction using TP drilling significantly reduced the revision risk. The anatomic reconstruction demands the use of the TP or outside-in femoral drilling technique which provides visualization of the native footprints and drilling of the femoral and tibial tunnels independent of each other [10, 19]. The development of anatomic reconstruction has led to questioning of the so long established transtibial (TT) technique since it has been shown to result in non-anatomic positioning of the ACL [14]. The impact of the surgical techniques utilized in a single-bundle reconstruction differs in terms of risk of revision and should thus be considered when comparisons with double-bundle reconstructions are made. A valuable tool when grading and evaluating anatomic ACL reconstructions is the Anatomic Anterior Cruciate Ligament Reconstruction Scoring Checklist (AARSC) [23]. The checklist includes 17 items of importance when performing an anatomic reconstruction where implementation of more items from the AARSC results in a more anatomic reconstruction.

The purpose of this study was to implement the AARSC to a register-based cohort in order to organize single-bundle reconstructions into homogenous groups based on the items fulfilled in the AARSC so that subsequently comparison of the revision rates between single- and double-bundle reconstructions could be made. To date, this is the first national population-based register study to compare single- versus double-bundle reconstruction when also implementing the AARSC. It was hypothesized that the double-bundle reconstruction would be associated with a lower revision frequency than single-bundle.

## Materials and methods

The Swedish National Knee Ligament Register (SNKLR) was used to extract patient data eligible for the study. Data from patients who underwent either single- or double-bundle ACL reconstruction during the period January 1, 2007–December 31, 2014 were included. Only patients aged 13 years or older with primary reconstruction using hamstring tendon autograft were considered eligible. Contralateral knee injury occurring during the follow-up period was not considered as an exclusion criterion. Full inclusion and exclusion criteria are summarized in Table 1. The date of primary ACL reconstruction marked the start of each patient’s follow-up period, which ended on 31 December 2014 or on the date of revision ACL surgery. Primary end point was set as ACL revision surgery. Patients having a possibly shorter follow-up than the earliest documented event (revision ACL surgery) in the specific cohort were censored from analysis, apart from that no minimum follow-up time was pre-specified. Data from individuals undergoing ACL revision surgery were included up until the date of their revision procedure; thus, the postoperative data from these patients was not included in analysis.

**Table 1:** Inclusion and exclusion criteria

Inclusion criteria
Primary ACL reconstruction
ACL reconstruction using hamstring tendon autograft
Single- or double-bundle ACL reconstruction
Exclusion criteria
Date of index surgery before January 1, 2007
Use of graft type other than hamstring tendon autograft
Age <13 years
Concomitant fracture
Concomitant nerve- or vessel injury
Concomitant ligament injury
Combined ACL reconstruction performed

### The Swedish National Knee Ligament Register

The SNKLR is a nationwide database established in January 2005, and 92.9% of all eligible units for ACL reconstruction in Sweden are linked to the register. Over 90% of the ACL reconstructions annually performed in Sweden are therefore registered [6, 15]. The register is sectioned in one data-registration for surgeons and one for patients. Information about activity at the time of injury, date of injury and surgery, fixation method and graft choice is reported. Concomitant injuries, and previous surgery to the knee or the contralateral knee are also registered along with information of all interventions made to the injured knee. Revision surgery or reoperation is separately registered, and the event is correlated with the primary ACL reconstruction. The patient reported outcome is evaluated through two questionnaires, the EQ-5D (European Quality of Life-5 Dimensions) and the KOOS (Knee injury and Osteoarthritis Outcome Score). The patients’ response rate preoperatively and at 1, 2 and 5 years range from 38 to 72% [15].

### Questionnaire

Through an online-based questionnaire, detailed information about surgical technique(s) performed by ACL surgeons in Sweden could be collected and matched to a specific ACL reconstruction, a method first described by Desai et al. [4]. The questionnaire was based on the items in the AARSC [23]. All the 175 surgeons registered in the SNKLR as of 31 December 2014 had the possibility to complete the questionnaire online between Jan 2015 and 30 April 2015. The response rate to the questionnaire was 61.7% [4]. The design of the questionnaire made it possible to identify which surgical technique(s) each surgeon performed during a specific period of time, thereby matching each patient’s ACL reconstruction to a specific technique.

### Organization of the ACL reconstructions into subgroups

The single-bundle reconstructions were sorted into groups defined by which surgical techniques had been implemented. In the present study, the same method and pre-defined groups were used as it was first described by Desai [4]. By combining eight different items from the AARSC, five groups were generated that were characterized by a specific combination of fulfilled items from the AARSC. The combinations of items were selected in order to produce homogenous groups with regard to surgical technique and size. Thus, for a specific ACL reconstruction to be placed in one of the groups the surgeon who performed the reconstruction must have answered to meet the requirements of the group. Each group has at least a few mandatory “yes” or “no” answers regarding the technique used, while some items are considered non-mandatory in some of the groups (Table 2). All single-bundle procedures meeting the inclusion criteria of the study could not be matched with a subgroup since not all surgeons responded to the survey and their surgical technique remains unknown. Furthermore, if a surgeon for any reason could not specify which techniques he or she implemented during a period of time, the reconstructions performed by that surgeon during that interval was not included in a subgroup. The limited amount of double-bundle reconstructions registered during the period of interest made it difficult to further subgroup these reconstructions. The double-bundle group was analysed as one unit, regardless of which surgical techniques had been implemented in the reconstructions included.

**Table 2:** Answer requirements characterizing defined groups

Group	Use of an Acc medial portal	Visualization of the femoral ACL insertion site	Visualization of the tibial ACL insertion site	Lateral intercondylar ridge identified	Bifurcate ridge identified	Placing the femoral tunnel(s) in the femoral ACL insertion site	Placing the tibial tunnel(s) in the tibial ACL insertion site	Transportal drilling of the femoral ACL tunnel(s)
TP reference	Yes	Yes	Yes	Yes	Yes	Yes	Yes	Yes
TP anatomic						Yes	Yes	Yes
TT anatomic						Yes	Yes	No
TT partial-anatomic						No	Yes	No
TT non-anatomic						No	No	No

Empty spaces are not assigned a mandatory answer requirement. Surgeons can thus answer “yes” or “no” to these items

*Acc* accessory, *TP* transportal, *TT* transtibial

This cohort study was conducted according to the WMA Declaration of Helsinki. Participation in the Swedish National Knee Ligament Register is voluntary for patients and surgeons. No written consent is necessary for national databases in Sweden. Investigators had access only to unidentifiable patient data. The study was approved by the Regional Ethics Committee in Gothenburg, Sweden (reference number: 760-14).

### Statistical analysis

Tables and diagrams were generated using Microsoft Excel for Windows (Version 14.0.7, Microsoft Corp, Redmond, Washington, USA). A statistician assigned to the SNKLR performed all statistical analyses. Statistical analysis was performed in IBM SPSS statistics (version 23.0, IBM Corp, Armonk, New York, USA). The data were summarized using counts and percentages for descriptive data and means ± SDs and median and range for patient-reported outcome data. The end point of revision surgery was analysed as time-to-event outcomes using Cox proportional hazards regression. Kaplan–Meier curves and log minus log plots were used to visually test the assumption of proportionality. All survival estimates and hazard ratios (HRs) were reported with 95% confidence intervals (CI). Statistical significance was defined as a 95% CI for hazard ratios not including 1.0, and alpha was set to 0.05. Additionally, a multivariate analysis adjusted for possible confounding factors (age and patient sex) was performed using a Cox proportional hazards regression expressed as HR and 95% CI.

## Results

**Fig. 1:**
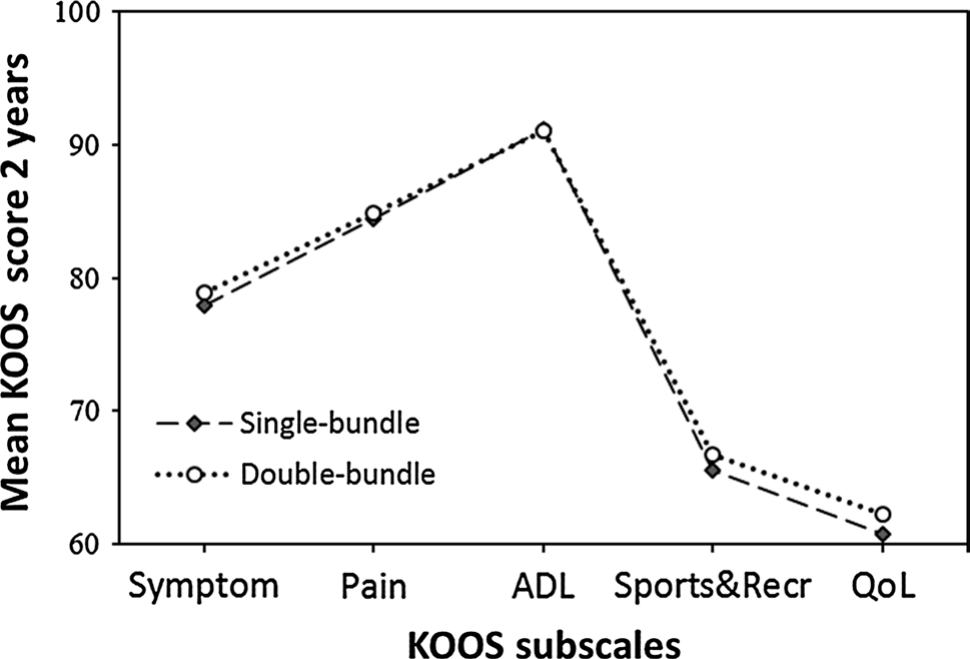
Mean KOOS scores 2 years after surgery for patients undergoing single-bundle and double-bundle ACL reconstruction, respectively. Please note the scale of the *y-axis* as the scale starts at a KOOS score value of 60. *KOOS* Knee injury and Osteoarthritis Outcome Score, *ADL* activities of daily living, *Recr* recreation, *QoL* quality of life

**Fig. 2:**
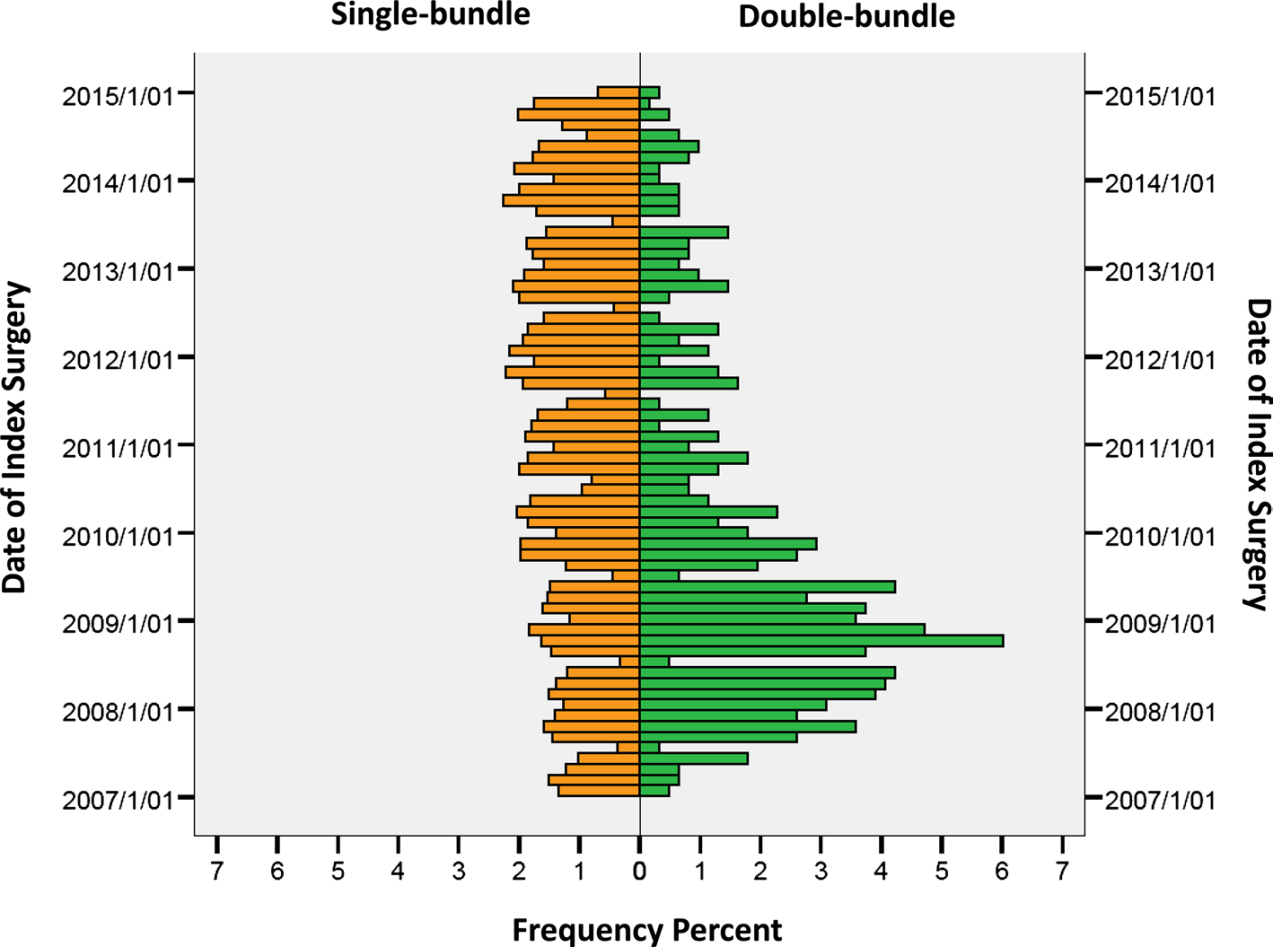
Distribution of date of index ACL surgery for all single-bundle and double-bundle procedures

A total of 22,460 patients were included in the study [12,777 (56.9%) males and 9683 (41.3%) females]. Patient characteristics are presented in Table 3. Mean 2-year postoperative KOOS score for single- and double-bundle, respectively, is found in Fig. 1. The distribution of the date of index ACL surgery for all single-bundle and double-bundle procedures expressed in per cent is presented in Fig. 2. In total 701 (3.1%) revision surgeries were reported between 2007 and 2014 (Table 4).

**Table 3:** Baseline patient characteristics

	Single-bundle	Double-bundle
Gender [*N* (%)]		
Male	12,401 (56.8)	376 (61.2)
Female	9445 (43.2)	238 (38.8)
Age at index ACL reconstruction [*N* (%)]		
13–15 years	1403 (6.4)	23 (3.7)
16–20 years	6120 (28.0)	165 (26.9)
21–25 years	4435 (20.3)	113 (18.4)
26–30 years	3064 (14.0)	97 (15.8)
31–35 years	2134 (9.8)	71 (11.6)
36–40 years	1910 (8.7)	73 (11.7)
41–45 years	1518 (6.9)	43 (7.0)
46–50 years	849 (3.9)	16 (2.6)
>51 years	413 (1.9)	14 (2.3)
Year of surgery [*N* (%)]		
2007	2183 (10.0)	78 (12.7)
2008	2380 (10.9)	191 (31.1)
2009	2586 (11.8)	136 (22.1)
2010	2884 (13.2)	70 (11.4)
2011	2896 (13.3)	40 (6.5)
2012	3072 (14.1)	39 (6.4)
2013	2.963 (13.6)	35 (5.7)
2014	2882 (13.2)	25 (4.1)

*ACL* anterior cruciate ligament

**Table 4:** Numbers of revision and risk of revision surgery for double-bundle, single-bundle and single-bundle subgroups

Groups		Crude model			Adjusted model^a^		
	No. of events	HR	95% CI	*p* value	HR	95% CI	*p* value
Double-bundle (reference) *n* = 614	12	1			1		
Single-bundle (*n* = 21,846)	689	2.12	1.20–3.76	0.010	1.98	1.12–3.51	0.019
*TP reference (n* = *5609*)	*146*	*2.08*	*1.15*–*3.75*	*0.015*	*1.87*	*1.04*–*3.38*	*0.037*
*TT non*-*anatomic (n* = *931)*	*28*	*1.42*	*0.72*–*2.79*	*n.s*	*1.23*	*0.63*–*2.43*	*n.s*
*TT anatomic (n* = *1717)*	*61*	*1.95*	*1.05*–*3.63*	*0.034*	*1.80*	*0.97*–*3.35*	*n.s*
*TT partial*-*anatomic (n* = *1013)*	*31*	*1.47*	*0.75*–*2.86*	*n.s*	*1.40*	*0.72*–*2.73*	*n.s*
*TP anatomic (n* = *3449)*	*133*	*2.66*	*1.47*–*4.81*	*0.001*	*2.51*	*1.39*–*4.54*	*0.002*
*Non*-*classified single*-*bundle (n* = *9127)*	*209*						

Numbers of revision and risk of revision surgery; single-bundle subgroups are presented in *italics*

*HR* hazard ratio, *CI* confidence interval, *TP* transportal, *TT* transtibial

^a^Cox regression analysis adjusted for patient gender and age at index ACL reconstruction

**Fig. 3:**
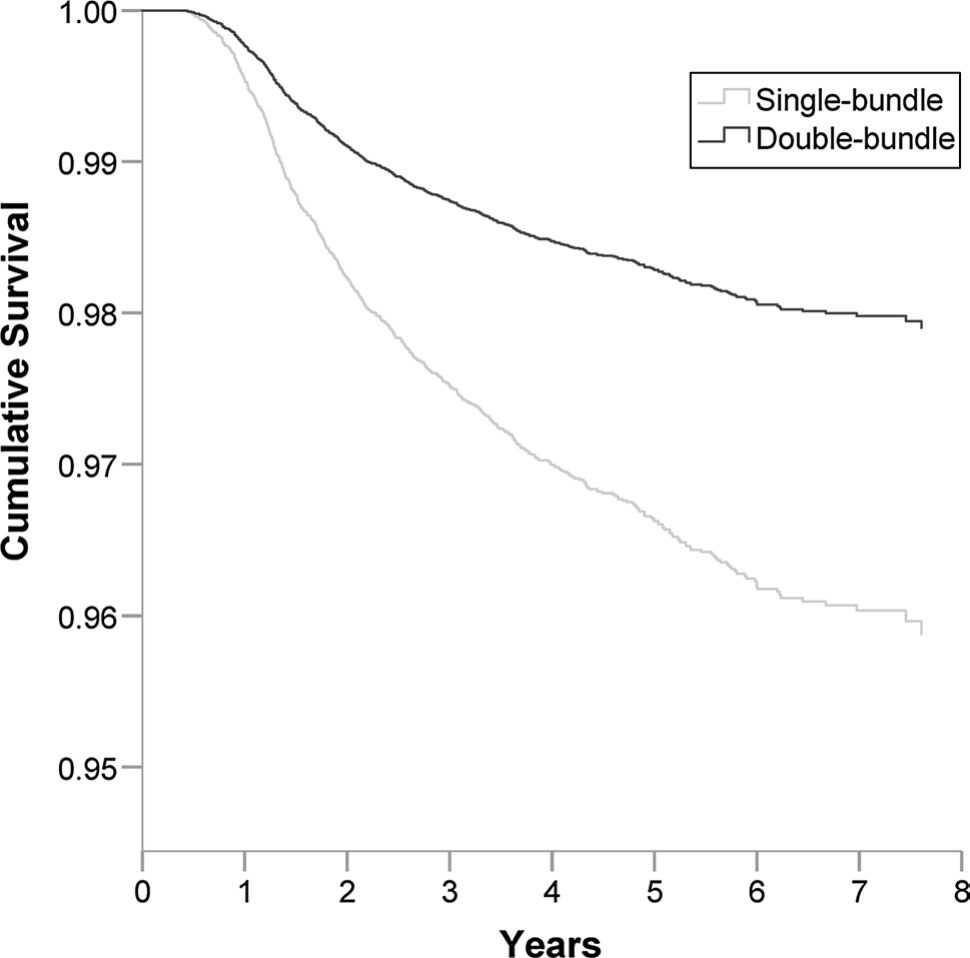
Cumulative survival function based on Cox proportional hazards regression of single-bundle, double-bundle and revision ACL surgery

**Fig. 4:**
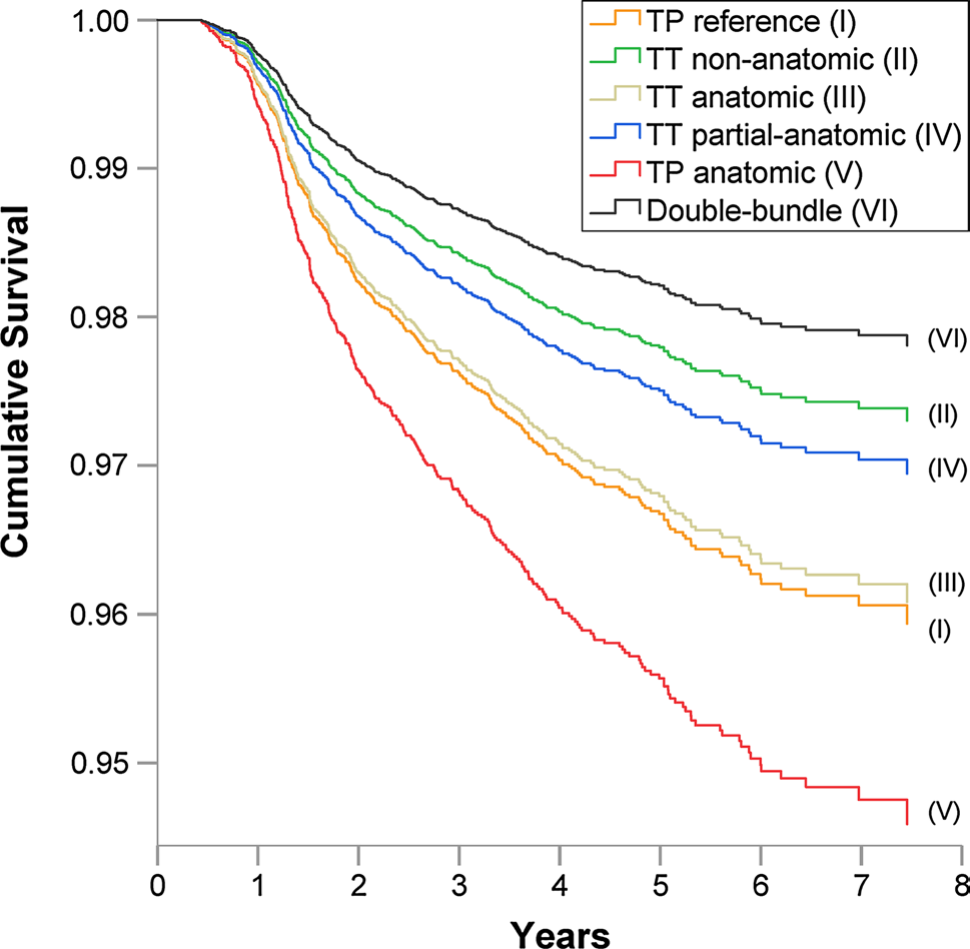
Cumulative survival function based on Cox proportional hazards regression of single-bundle subgroups, double-bundle and revision ACL surgery. The survival of each single-bundle subgroup is presented by a *separate curve*

There was an increased risk of revision surgery for patients who underwent index ACL surgery with single-bundle compared with double-bundle [HR 1.98 (95% CI 1.12–3.51), *p* = 0.019] adjusted for gender and age at index surgery (Table 4; Fig. 3). In the subgroup analysis, the double-bundle had the highest cumulative survival rates of all groups followed by TT non-anatomic, while the lowest was found in TP anatomic group (Fig. 4). The risk of revision surgery was significantly increased in patients undergoing single-bundle with TP anatomic technique [adjusted HR 2.51 (95% CI 1.39–4.54), *p* = 0.002], and TP reference surgical technique [adjusted HR 1.87 (95% CI 1.04–3.38), *p* = 0.037], compared to double-bundle (Table 4). No significant difference in terms of risk of revision was seen in the other single-bundle subgroups when compared with double-bundle.

## Discussion

The most important finding of present study was that double-bundle reconstruction was associated with a significant lower risk of revision surgery compared with single-bundle reconstruction, confirming the hypothesis of the study. Furthermore, the study showed a distinction in the risk of revision surgery among single-bundle procedures depending on the surgical techniques implemented.

Previous studies of single- versus double-bundle reconstruction often include a combined analysis of anatomic and non-anatomic techniques, and surgical data of the two techniques are grossly underreported in clinical studies [5, 25, 26]. This study is unique in the sense that it compared the revision rates of single- and double-bundle reconstruction in a large population-based cohort when also taking several surgical variables into consideration. The TP reference group comprised the most anatomic single-bundle reconstructions since all eight items selected from the AARCS were fulfilled. The TP anatomic group was characterized by implementation of only three mandatory techniques. In concordance with previous findings by Desai [4], this study showed that a TP reference technique, defined in terms of a more complete anatomic reconstruction, reduces the risk of revision surgery considerably when the TP drilling technique is utilized. Based on the closely overlapping confidence intervals of the TP reference and the TT anatomic subgroups, the two techniques may be considered equal in terms of risk of revision compared with double-bundle, which is also reported in previous study by Desai [4].

An anatomically placed graft is exposed to higher graft forces [1, 11, 16] which might predispose graft failure and explain the higher revision frequency among anatomic reconstructions. However, the double-bundle reconstruction more closely resembles the native anatomy where two separately tensioned bundles result in a more naturally distributed graft load during knee range of motion compared with an anatomic single-bundle reconstruction. Along with the greater proportion of femoral and tibial insertion sites covered by the double-bundle, these factors could have positive influence on incorporation, vascularization and maturation of the grafts and explain the lower revision frequency of the double-bundle. By non-anatomic reconstruction the graft load decrease, although several studies have shown that this is done at the expense of rotational stability and function [11, 12, 27]. Following this reasoning, it is worth considering that a double-bundle may have a potential to both distribute graft load properly and provide rotational stability. The ability to better control rotational forces may also directly prevent traumatic re-rupture by counteracting excessive rotational movements of the knee joint.

With today’s knowledge about the superiority of the double-bundle reconstruction in terms of restoration of knee joint laxity and the ambition to individualize the procedure for each patient [10], it is likely that patients selected for double-bundle reconstruction have high activity-level demands, great rotational instability and a large size of the knee joint. Assuming this, it is remarkable that only 12 revision surgeries were registered in the group. Furthermore, the double-bundle reconstructions included in this study were performed during a time when the technique was still new to many of the surgeons. The yearly decrease in numbers of double-bundle procedures performed in Sweden may reflect that the procedure is technically challenging, time-consuming and associated with a steep learning curve [17]. It must be emphasized that a revision surgery of a double-bundle reconstruction is more complicated than that of a single-bundle reconstruction [8, 9]. This fact enhances the possibility that some revision surgeries are not performed in the double-bundle group even though it might be indicated which may result in misleadingly low revision rates in the group. Björnsson et al. [2] have previously investigated the difference in revision rates between single- and double-bundle reconstructions in the SNKLR. Patients undergoing primary ACL reconstruction between 2005 and 2011 were included. In contrast to the present study, no significant differences were found in revision rates between the groups. However, present study includes a much larger patient cohort and a longer follow-up period.

There are some limitations to this study. First, the limited numbers of double-bundle procedures performed produce large differences in volume of data between the single- and double-bundle cohorts. Consequently, the double-bundle group could not be divided into subgroups with regard to the surgical technique. To do so would have been desirable in order to fully adopt the method of comparing similar surgical techniques with each other. The subgrouping in this study is based on the response-frequency to the questionnaire by the surgeons. There is a risk that reconstructions which may have influenced the analysis of a subgroup were not included in it since their surgical technique was unknown due to non-responding surgeons. The risk of recall bias must be noted as the reliability of the survey depends on correctly reported information about which surgical technique(s) the surgeon implemented during a specific period of time. Also, the primary outcome of revision surgery might withhold the true occurrence of graft failure, and the SNKLR does not provide information about for example patient characteristic, postoperative rehabilitation programme or postoperative activity level which all may influence the outcome of an ACL reconstruction.

Regarding the clinical setting, this study indicates that double-bundle reconstruction is a satisfactorily option and should be considered in the same extent as single-bundle when individualizing treatment. Furthermore, surgeons performing TP drilling should always intend to do so in a thorough way with regard to anatomic technique.

## Conclusions

Double-bundle ACL reconstruction is associated with a lower risk of revision surgery than single-bundle ACL reconstruction. Single-bundle procedures performed using transportal femoral drilling technique had significantly higher risk of revision surgery compared with double-bundle. However, a reference reconstruction with transportal drilling defined as a more complete anatomic reconstruction reduces the risk of revision surgery considerably. The lower revision frequency of the double-bundle reconstruction found in this study indicates the need for further development, utilization and evaluation of this technique.
